# Effect of cardiac rehabilitation on circulating microRNA expression in heart failure: a preliminary study

**DOI:** 10.20407/fmj.2020-010

**Published:** 2020-11-13

**Authors:** Ryo Yamada, Satoshi Okumura, Yuji Kono, Akane Miyazaki, Yudai Niwa, Takehiro Ito, Sayano Ueda, Tomoya Ishiguro, Masataka Yoshinaga, Wakaya Fujiwara, Mutsuharu Hayashi, Yukio Ozaki, Eiichi Saitoh, Hideo Izawa

**Affiliations:** 1 Department of Cardiology, Fujita Health University Bantane Hospital, Nagoya, Aichi, Japan; 2 Department of Rehabilitation, Fujita Health University Bantane Hospital, Nagoya, Aichi, Japan; 3 Department of Cardiology, Fujita Health University, School of Medicine, Toyoake, Aichi, Japan; 4 Department of Rehabilitation Medicine I, Fujita Health University, School of Medicine, Toyoake, Aichi, Japan

**Keywords:** MicroRNA, Heart failure, Cardiac rehabilitation, Exercise

## Abstract

**Objectives:**

There are benefits of exercise-based cardiac rehabilitation (CR) in patients with heart failure (HF), but their underlying molecular mechanisms remain elusive. The effect of CR on the expression profile of circulating microRNAs (miRNAs), which are short noncoding RNAs that regulate posttranscriptional expression of target genes, is unknown. If miRNAs respond to changes following CR for HF, then serum profiling of miRNAs may reveal cardioprotective mechanisms of CR.

**Methods:**

This study enrolled three hospitalized patients with progressed systolic HF and three normal volunteer controls. In patients, CR was initiated after improvement of HF, which included 2 weeks of bicycle ergometer and resistance exercises. Genome-wide expression profiling of circulating miRNAs was performed using microarrays for the patients (mean±SD age, 60.0±12.2 years) and controls (58.7±0.58 years). Circulating miRNA expression profiles were compared between patients with HF before and after CR and the controls.

**Results:**

Expression levels of two miRNAs were significantly different in patients before CR compared with controls and patients after CR. The expression of hsa-miR-125b-1-3p was significantly downregulated and that of hsa-miR-1290 was significantly upregulated in patients before CR.

**Conclusions:**

When performing CR, expression of certain circulating miRNAs in patients with HF is restored to nonpathological levels. The benefits of CR for HF may result from regulation of miRNAs through multiple effects of gene expression.

## Introduction

Cardiac rehabilitation (CR) that includes exercise training results in a broad range of positive effects for patients with cardiac diseases. On the basis of strong evidence, CR is recommended for patients with heart failure (HF).^1^ In patients with mild to moderate chronic HF, exercise training improves their exercise tolerance and quality of life, as well as having the potential to reduce mortality and hospitalization.^2^ However, this recommendation is not sufficiently implemented in clinical practice. Clarification of the molecular mechanisms underlying the effect of CR could assist in an understanding of the benefit of CR, and lead to an increase in awareness of CR in clinical practice.

MicroRNAs (miRNAs) are a family of single-stranded, short, non-coding RNA sequences that comprise 19–23 nucleotides. These miRNAs act as important posttranscriptional regulators of genetic expression through translational repression or transcript cleavage resulting from binding to the target mRNA.^3–5^ The miRNAs were first reported as endogenous mediators in the worm *Caenorhabditis elegans*.^6^ Since this time, miRNAs have been shown to be involved in regulating various cellular processes, including cellular differentiation, proliferation, and apoptosis.^7^ A lot of evidence of the crucial roles of miRNAs in various diseases has accumulated over recent years,^8–10^ but their significance in molecular biological processes of cardiac diseases are only beginning to be recognized.^11,12^

The miRNAs exist in a stable form in the bloodstream and are referred to as circulating miRNAs. Circulating miRNAs could become useful biomarkers for various diseases,^13,14^ including cardiac diseases.^15,16^ The present study aimed to investigate whether the cardioprotective mechanisms of CR for patients with HF involve changes in circulating miRNAs.

## Materials and Methods

### Subjects, CR program, and blood sampling

Three patients with HF and three healthy controls were enrolled in the study. The patients were hospitalized for treatment of progressed HF with systolic left ventricular dysfunction from New York Heart Association classes I–II. The controls were volunteers who had no previous medical history and were recruited from staff at Fujita Health University Bantane Hospital. Written informed consent was obtained from all of the subjects and the protocol was approved by the Research Ethics Committee of Fujita Health University (HM16-203). In patients with HF, CR was initiated after recovery from New York Heart Association classes II to I by 1 week of treatment for HF. The CR exercise program comprised a bicycle ergometer at an anaerobic threshold intensity that was determined from a cardiopulmonary exercise test and resistance training for 20 min twice a day, 5 days a week. This exercise was performed daily for 2 weeks while the patient was hospitalized. Microarray analysis of circulating miRNAs was performed for these three patients before and after the CR program, as well as for the three controls. Blood samples were obtained in the morning in a calm environment, with the subject in a fasted state. A 20-mL aliquot of blood was drawn from a peripheral vein into a spitz tube containing serum separating agent. The serum was isolated by centrifugation at 3000 rpm for 10 min, transferred into an Eppendorf tube, and stored at –90°C.

### RNA extraction and miRNA expression profiling

Total RNA was isolated from the serum using 3D-Gene RNA extraction reagent (Toray Industries, Kamakura, Japan) according to the manufacturer’s instructions. The extracted RNA was checked with a Bioanalyzer (Agilent, Santa Clara, CA, USA) and labeled with a 3D-Gene circulating miRNA labeling kit (Toray Industries). The labeled RNAs were hybridized onto a 3D-Gene Serum miRNA Oligo chip (Toray Industries). We ensured that the annotation and oligonucleotide sequences of the probes conformed with the miRBase miRNA database (miRbase.org). After stringent washing, the fluorescent signals were scanned with a 3D-Gene Scanner (Toray Industries) and analyzed using 3D-Gene Extraction software (Toray Industries). The raw data for each spot were normalized by substitution with the mean intensity of the background signal, which was determined from the 95% confidence intervals for the signal intensities of all of the brank spots. Measurements of spots were considered to be valid when the signal intensities were >2 standard deviations (SDs) from the background signal intensity. Relative expression levels of miRNAs were calculated by comparison with the signal intensities of the valid spots throughout the microarray analysis. The data were then globally normalized for each array by adjusting the median of the signal intensity to 25. The miRNA results for the patients before CR were compared with those of the controls and those of the patients after CR.

### Statistical analysis

Continuous variables are presented as the mean±SD, and categorical variables are presented as numbers and percentages. Differences in the characteristics between patients with HF and controls were evaluated with unpaired *t* tests for continuous variables and chi-square analysis or Fisher’s exact test for absolute categorical variables. Differences in the characteristics between patients with HF before and after CR were evaluated with the paired t test for continuous variables. Statistical significance was set at p<0.05. In the microarray analyses, significant differential expression was defined by a mean fold difference of >2 or <0.5 relative to the controls or the patients after CR, with a p value adjusted by an FDR <0.05. All statistical analyses were performed with IBM SPSS statistics version 20.0 (IBM Corporation, Armonk, NY, USA).

## Results

### Subjects’ characteristics

The clinical characteristics of the patients with HF and the controls are shown in Table 1. The patients with HF and the controls were all men, and there was no significant difference in age between the patients and controls. Patients with HF had significantly higher levels of N-terminal pro-brain natriuretic peptide compared with controls. Table 2 shows comparison of the characteristics of the patients with HF before and after CR. Patients after CR had a significantly lower systolic blood pressure and were more likely to have a lower body weight, body mass index, diastolic pressure, and heart rate compared with patients before CR.

### Circulating miRNA expression in HF

**Figure 1:**
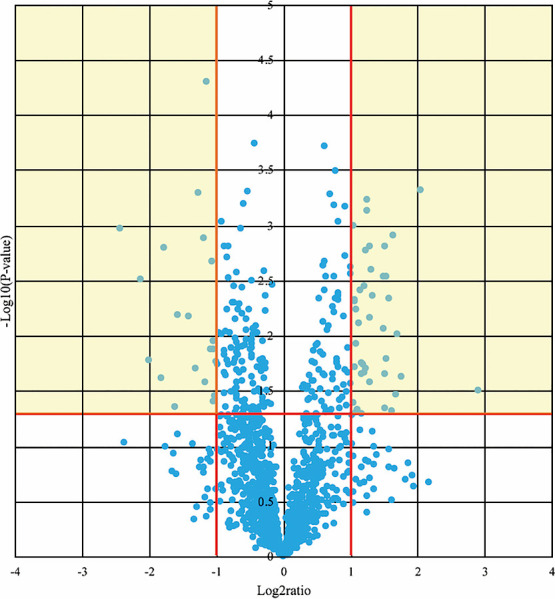
Volcano plot showing the differences in circulating miRNA expression in controls compared with patients with heart failure before cardiac rehabilitation. The x and y axes indicate the signal difference (log_2_ ratio) and the level of statistical significance (–log_10_ p value), respectively. The vertical red lines correspond to fold changes of 2.0 and 0.5, and the horizontal red line represents a p value of 0.05. The points in the yellow areas represent the miRNAs that met the criteria for significant differential expression.

The p values and fold differences for all of the circulating miRNAs were compared between patients before CR and controls and are plotted in a volcano plot in Figure 1. Of these, 61 miRNAs met the criteria for a significant difference between patients before CR and controls. Expression levels of 41 miRNAs were significantly lower and those of 20 miRNAs were significantly higher in patients before CR compared with controls (Table 3).

### Circulating miRNA expression after CR

**Figure 2:**
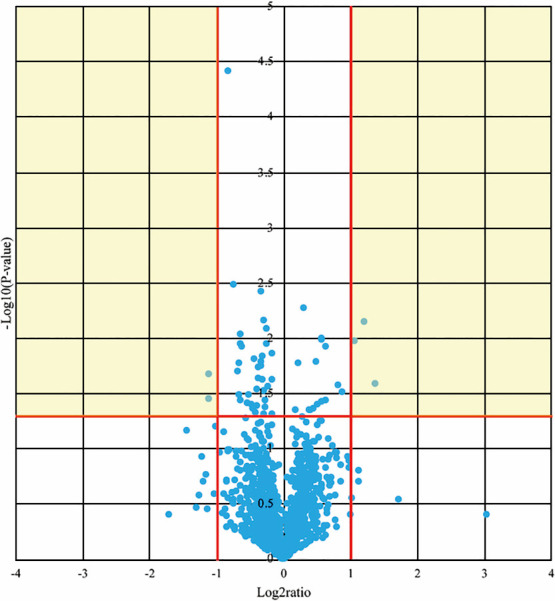
Volcano plot showing the differences in circulating miRNA expression in patients with heart failure after cardiac rehabilitation compared with those in patients before cardiac rehabilitation. The x and y axes indicate the signal difference (log_2_ ratio) and the level of statistical significance (–log_10_ p value), respectively. The vertical red lines correspond to fold differences of 2.0 and 0.5, and the horizontal red line represents a p value of 0.05. The points in the yellow areas represent the miRNAs that met the criteria for significant differential expression.

The p values and fold differences for all of the circulating miRNAs were compared between before and after CR in patients with HF and are plotted in a volcano plot in Figure 2. Of these, five miRNAs met the criteria for a significant difference between before and after CR in patients with HF. Expression levels of three miRNAs were significantly lower and those of two miRNAs were significantly higher in patients before CR compared with patients after CR (Table 4).

When these results of the two volcano plots were combined, expression of two miRNAs was significantly different in patients before CR compared with controls and patients after CR as follows. Expression of hsa-miR-125b-1-3p was significantly downregulated and that of hsa-miR-1290 was significantly upregulated in patients before CR. Additionally, hsa-miR-24-3p and hsa-miR-3661 showed a trend of downregulation in patients before CR compared with controls and patients after CR (fold difference from 0.5–2.0, p<0.05), and hsa-miR-30c-1-3p, hsa-miR-196b-3p, hsa-miR-3945, and hsa-miR-7151-3p showed a trend of upregulation.

## Discussion

This study used genome-wide microarray analyses to analyze expression levels of circulating miRNAs in three patients with HF before and after undergoing a 2-week program of CR in hospital. Our study showed for the first time that expression levels of two circulating miRNAs were significantly different before and after CR for HF. Before CR, miR-125b expression was significantly downregulated and miR-1290 expression was significantly upregulated compared with controls, and both of these miRNA levels returned to levels of controls after CR. These findings suggest that CR normalizes altered expression levels of some miRNAs in patients with HF.

### Circulating miRNAs and exercise training

Circulating miRNAs in human serum and plasma were first identified in approximately 2008.^17,18^ The miRNAs are transported from the nucleus into the cytoplasm, where they become associated with various proteins and are secreted into the bloodstream. As well as circulating in association with proteins, miRNAs can be packaged into extracellular vesicles or high-density lipoproteins or released in apoptotic bodies. Circulating miRNAs are absorbed by remote tissues, where they regulate gene expression by translational repression, mRNA degradation, and deadenylation of mRNAs.^19,20^ Circulating miRNAs are secreted from various tissues, including the endothelium, in response to exercise. One potential mechanism underlying the change in miRNA profile with exercise is that increased shear stress along the endothelium may stimulate secretion of specific miRNAs from endothelial cells. The miRNAs miR-21, -126, -146a, and -210 are among the best described circulating miRNAs associated with the response to exercise in healthy men, and these are enriched in the endothelium.^21^ In patients with HF, circulating miR-146a levels do not change following acute exercise, whereas circulating miR-940 enriched in the myocardium is increased compared with healthy individuals.^22^ Therefore, the response of the circulating miRNA profile to exercise training in patients with HF requires careful evaluation to assess the differences to responses of healthy individuals. To the best of our knowledge, there have been no previous reports of changes in the profile of circulating miRNAs following CR that includes exercise training in patients with HF.

### miRNAs associated with CR for HF

The miR-125 family has been implicated in various carcinomas and other diseases as either repressors or promoters.^23–25^ Members of this family play crucial roles in various cellular processes, including cell differentiation, proliferation, and apoptosis, by targeting many transcription factors,^26^ matrix metalloproteinase,^27,28^ and growth factors.^29^ miR-125 acts in different ways according to the cell context, such as playing important roles in the mitochondrial apoptosis pathway by targeting pro-apoptosis or anti-apoptosis genes.^30^ The pathological mechanisms of circulating miR-125 in the human heart have not been determined, although miR-125 was found to be upregulated in fibrotic heart tissue.^31^ In the present study, circulating miR-125b levels were significantly lower in patients with HF compared with controls and increased to normal levels after CR. Although the relationship between expression levels of miR-125 in cardiomyocytes and the blood has not been established, this result suggests that CR exerts protection against HF through changes in circulating and myocardial miR-125 expression levels.

miR-1290 plays a major role in initiation and progress of cancer. Studies have shown that increased miR-1290 levels are associated with proliferation, invasion, metastasis, and the clinical stage of cancer.^32–34^ A feature of the tumor microenvironment is regional severe hypoxia.^35^ Therefore, a potential role of miR-1290 is regulation of cell survival under hypoxic conditions. Recently, Wu et al.^36^ reported that the molecular mechanism underlying increased survival of cardiomyocytes with asiatic acid might be the negative relationship between expression levels of miR-1290 and the transcription factor hypoxia-inducible factor 3A in cardiomyocytes. A previous report showed that patients with HF who underwent CR improved their exercise tolerability.^37^ The relationship between expression levels of circulating and myocardial miR-1290 has not been clarified. However, our finding that circulating miR-1290 expression was significantly higher in patients before CR compared with controls with a decrease after CR suggests that miR-1290 contributes to increased exercise tolerability following CR through its molecular biological role of boosting resistance to cellular hypoxia.

In the present study, expression levels of circulating miR-24-3p, miR-196b, and miR-30c showed a tendency to be different in patients before CR compared with controls and patients after CR. Previous studies have reported interesting findings for the biological roles of these miRNAs. Qian et al.^38^ showed that miR-24 inhibited cardiomyocyte apoptosis in a mouse myocardial infarction model. Dahlmans et al.^39^ reported a correlation between miR-196b levels and *in vivo* mitochondrial function in human skeletal muscle. Duisters et al.^40^ reported that miR-30 directly downregulated connective tissue growth factor, a key molecule in the process of fibrosis, thereby regulating structural changes in the extracellular matrix of the myocardium. These findings are consistent with the positive effect of CR, such as reverse left ventricular remodeling,^41^ improvement of mitochondrial dysfunction in skeletal muscle,^42^ and a reduction in cardiac fibrosis.^43^

### Clinical implications and limitations

The present results help to identify aspects of the biological mechanism of CR in patients with HF, such as improvement of mitochondrial dysfunction, an increased cellular resistance to hypoxic conditions, and a reduction in cardiomyocyte apoptosis and cardiac fibrosis. However, our study has several limitations. First, the number of subjects was small. Potential variation in circulating miRNAs among the three patients could have affected the results of the present study. Second, the possibility that treatment for HF affected the circulating miRNA expression profile cannot to be excluded, such as body weight loss and a decrease in blood pressure. Finally, the relationships between expression levels of circulating and intracellular miRNAs are unclear. However, to the best of our knowledge, this is the first study to evaluate the change in circulating miRNA profiles resulting from CR in patients with HF. The present results support the beneficial effect of CR for HF and could be the biological findings that enhance the evidence of CR. A large-scale study with polymerase chain reaction analysis is required to further investigate the roles of miRNAs in CR for HF at a genetic level.

## Conclusion

In patients with HF, CR can restore expression of certain circulating miRNAs to nonpathological levels. Regulation of expression of multiple genes by these restored miRNAs may contribute to the beneficial effects of CR on HF.

## Figures and Tables

**Table1:** Clinical characteristics of patients with heart failure before cardiac rehabilitation and controls

Characteristics	Patients (n=3)	Controls (n=3)	p
Age (y)	60.0±12.2	58.7±0.6	0.57
Male sex	3 (100%)	3 (100%)	—
BMI (kg/m^2^)	31.0±12.6	22.4±2.2	0.59
Pervious medical history			
Hypertension	1 (33%)	0 (0%)	—
Dyslipidemia	1 (33%)	0 (0%)	—
Diabetes	0 (0%)	0 (0%)	—
Smoking	2 (66%)	0 (0%)	—
Medication			
Diuretics	3 (100%)	0 (0%)	—
ACEIs/ARBs	3 (100%)	0 (0%)	—
Beta-blockers	3 (100%)	0 (0%)	—
Hemoglobin (mg/dL)	14.0±0.5	15.3±1.3	0.13
Creatinine (mg/dL)	0.85±0.23	0.89±0.12	0.85
eGFR (mL/min/1.73 m^2^)	76.7±23.8	69.2±10.2	0.85
NT-pro BNP (pg/mL)	2489±2015	52.7±44.1	0.02

ACEI, angiotensin-converting enzyme inhibitor; ARB, angiotensin receptor blocker; BMI, body mass index; eGFR, estimated glomerular filtration rate; NT-pro BNP, N-terminal pro-brain natriuretic peptide.

**Table2:** Characteristics of the three selected patients with heart failure before and after CR

Characteristics	Before CR	After CR	p
Body weight (kg)	88.7±44.8	74.6±30.1	0.24
BMI (kg/m^2^)	31.0±12.6	26.2±8.0	0.22
Systolic BP (mmHg)	142.7±4.0	102.7±2.5	<0.001
Diastolic BP (mmHg)	84.0±23.6	66.0±17.5	0.10
Heart rate (bpm)	78.3±28.0	60.7±6.4	0.30
Hemoglobin (mg/dL)	14.0±0.5	15.6±1.6	0.31
Creatinine (mg/dL)	0.85±0.23	0.89±0.26	0.32
eGFR (mL/min/1.73 m^2^)	76.7±23.8	73.7±24.2	0.20
NT-pro BNP (pg/mL)	2489±2015	1023±926	0.15
Cardiac ultrasonography			
LVDd (mm)	60.6±7.4	63.4±7.5	0.38
LVDs (mm)	50.9±6.3	50.7±3.1	0.96
LVEF (%)	40.0±9.2	46.0±5.2	0.15

BMI, body mass index; BP, blood pressure; CR, cardiac rehabilitation; eGFR, estimated glomerular filtration rate; LVDd, left ventricular diastolic diameter; LVDs, left ventricular systolic diameter; LVEF, left ventricular ejection fraction; NT-pro BNP, N-terminal pro-brain natriuretic peptide.

**Table3:** Circulating miRNAs that showed significantly different expression levels in patients with heart failure before cardiac rehabilitation compared with controls

miRNAs	Regulation	miRNAs	Regulation
hsa-miR-124-3p	Downregulated	hsa-miR-1240	Upregulated
hsa-miR-125a-3p	Downregulated	hsa-miR-1285-3p	Upregulated
*hsa-miR-125b-1-3p*	Downregulated	*hsa-miR-1290*	Upregulated
hsa-miR-1470	Downregulated	hsa-miR-135a-3p	Upregulated
hsa-miR-151a-3p	Downregulated	hsa-miR-30b-3p	Upregulated
hsa-miR-184	Downregulated	hsa-miR-30c-1-3p	Upregulated
hsa-miR-3155a	Downregulated	hsa-miR-3127-5p	Upregulated
hsa-miR-3680-3p	Downregulated	hsa-miR-3945	Upregulated
hsa-miR-3714	Downregulated	hsa-miR-4271	Upregulated
hsa-miR-3925-5p	Downregulated	hsa-miR-4673	Upregulated
hsa-miR-3936	Downregulated	hsa-miR-4698	Upregulated
hsa-miR-4299	Downregulated	hsa-miR-4733-3p	Upregulated
hsa-miR-4300	Downregulated	hsa-miR-5096	Upregulated
hsa-miR-4443	Downregulated	hsa-miR-6081	Upregulated
hsa-miR-4444	Downregulated	hsa-miR-665	Upregulated
hsa-miR-4448	Downregulated	hsa-miR-6736-3p	Upregulated
hsa-miR-4530	Downregulated	hsa-miR-6812-3p	Upregulated
hsa-miR-4538	Downregulated	hsa-miR-7151-3p	Upregulated
hsa-miR-4635	Downregulated	hsa-miR-7641	Upregulated
hsa-miR-4681	Downregulated	hsa-miR-92a-2-5p	Upregulated
hsa-miR-4727-3p	Downregulated		
hsa-miR-4755-3p	Downregulated		
hsa-miR-4771	Downregulated		
hsa-miR-4793-5p	Downregulated		
hsa-miR-520d-5p	Downregulated		
hsa-miR-520e	Downregulated		
hsa-miR-524-5p	Downregulated		
hsa-miR-525-5p	Downregulated		
hsa-miR-551b-5p	Downregulated		
hsa-miR-575	Downregulated		
hsa-miR-593-5p	Downregulated		
hsa-miR-614	Downregulated		
hsa-miR-6501-3p	Downregulated		
hsa-miR-6507-5p	Downregulated		
hsa-miR-6509-5p	Downregulated		
hsa-miR-6717-5p	Downregulated		
hsa-miR-6892-5p	Downregulated		
hsa-miR-7154-3p	Downregulated		
hsa-miR-920	Downregulated		
hsa-miR-933	Downregulated		

**Table4:** Circulating miRNAs that showed significantly different expression levels in patients with heart failure before cardiac rehabilitation compared with patients with heart failure after cardiac rehabilitation

miRNAs	Regulation	miRNAs	Regulation
*hsa-miR-125b-1-3p*	Downregulated	*hsa-miR-1290*	Upregulated
hsa-miR-200c-3p	Downregulated	hsa-miR-196b-3p	Upregulated
hsa-miR-3181	Downregulated		

miRNA, microRNA.
